# Taurine Supplementation as a Neuroprotective Strategy upon Brain Dysfunction in Metabolic Syndrome and Diabetes

**DOI:** 10.3390/nu14061292

**Published:** 2022-03-18

**Authors:** Zeinab Rafiee, Alba M. García-Serrano, João M. N. Duarte

**Affiliations:** 1 Department of Experimental Medical Science, Faculty of Medicine, Lund University, 22100 Lund, Sweden; zeinab.rafiee@med.lu.se (Z.R.); albags89@gmail.com (A.M.G.-S.); 2 Wallenberg Centre for Molecular Medicine, Lund University, 22100 Lund, Sweden

**Keywords:** 2-aminoethanesulfonic acid, neurodegeneration, brain metabolism, diabetes, obesity

## Abstract

Obesity, type 2 diabetes, and their associated comorbidities impact brain metabolism and function and constitute risk factors for cognitive impairment. Alterations to taurine homeostasis can impact a number of biological processes, such as osmolarity control, calcium homeostasis, and inhibitory neurotransmission, and have been reported in both metabolic and neurodegenerative disorders. Models of neurodegenerative disorders show reduced brain taurine concentrations. On the other hand, models of insulin-dependent diabetes, insulin resistance, and diet-induced obesity display taurine accumulation in the hippocampus. Given the possible cytoprotective actions of taurine, such cerebral accumulation of taurine might constitute a compensatory mechanism that attempts to prevent neurodegeneration. The present article provides an overview of brain taurine homeostasis and reviews the mechanisms by which taurine can afford neuroprotection in individuals with obesity and diabetes. We conclude that further research is needed for understanding taurine homeostasis in metabolic disorders with an impact on brain function.

## 1. Introduction

**Figure 1:**
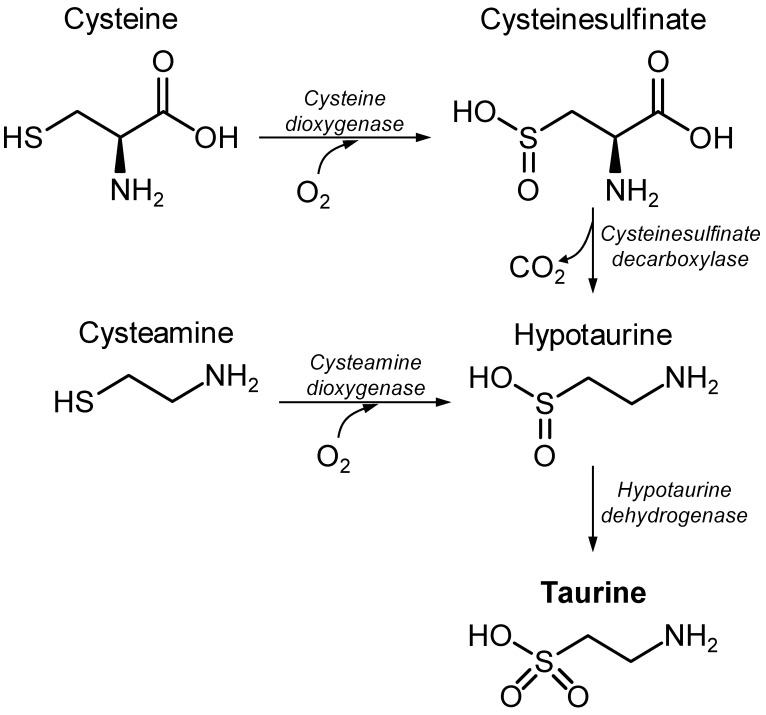
Synthesis of taurine in mammals from the sulfur amino acid cysteine.

Taurine, or 2-aminoethanesulfonic acid, was first isolated from ox bile in 1827, by Friedrich Tiedemann and Leopold Gmelin. Taurine is obtained from the diet or results from de novo synthesis through catabolism of the amino acid cysteine (Figure 1). Together with glycine, taurine is well known for bile acid amidation, producing bile salts for excretion. Taurine supplementation has been suggested to have beneficial effects on a number of disorders, for example, hypertension [1,2], congestive heart failure [3], ischemia–reperfusion myocardial injury [4], intracerebral hemorrhage [5], pulmonary fibrosis [6], obesity-induced low-grade inflammation [7]. The neuroprotective effects of taurine have received considerable attention, and there is a plethora of publications showing the ability of exogenously added taurine to prevent toxicity in neurons or astrocytes in vitro, as well as in animal models of neurological disorders (reviewed by Jakaria et al. [8]). Namely, taurine treatments have been shown to protect tissues and cells against oxidative stress (e.g., [9]), mitochondrial stress (e.g., [10]), or inflammation (e.g., [11]). In addition, brain taurine is known as an osmoregulator and neuromodulator [12,13] and is involved in numerous processes, such as the modulation of neuronal excitability, the cerebral control of the cardiorespiratory system, appetite regulation, resistance to hypoxia, osmoregulation, and anti-oxidation [14]. Enzymes that synthetize taurine show low activity in cats, dogs, and foxes, which develop pathologies when fed a taurine-deficient diet, namely, cardiomyopathy and myocardial dysfunction, retinal degeneration, neurological abnormalities, weakened immune response, pregnancy and fetal development complications, as well as gastrointestinal problems (see [15] and references therein). This is clear evidence advocating for the importance of taurine.

**Figure 2:**
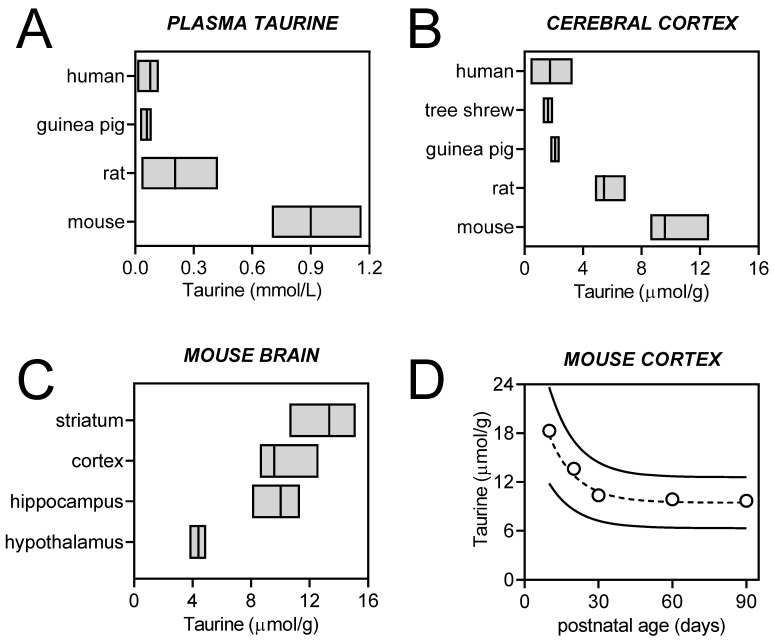
Concentrations of taurine in the plasma (**A**) and cerebral cortex of various species (**B**), in different areas of the mouse brain (**C**), and in the mouse cortex during development (**D**). Plasma taurine levels are indicated as mean and range for humans [2,20,21,22,23,24,25,26,27], guinea pigs [28,29], rat [30,31,32,33,34,35,36], and mice [37,38,39,40,41]. The plotted brain taurine concentration ranges are based on the concentrations reported in ^1^H MRS studies for humans [42,43,44,45,46,47], tree shrews [48], guinea pigs [49], Sprague–Dawley rats [50,51,52,53,54,55], and C57BL/6J mice [38,56,57,58,59,60].

Taurine is one of the most abundant metabolites in the central nervous system (CNS), whose levels show substantial variations across species, brain areas, and developmental stages (Figure 2). The particularly high concentration of taurine in the developing brain further suggests its developmental importance. Indeed, a relation between plasma taurine and neurodevelopment has been proposed [16]. This role of taurine in CNS development was made clear by experiments on cats fed a taurine-deficient diet [17]. More recent research proposes that taurine has neurotrophic effects, playing an important role in neurite outgrowth, synaptogenesis, and synaptic transmission during the early stages of brain development [18,19].

## 2. Taurine Homeostasis

Dietary taurine is absorbed by the gut, released into the blood stream, and excreted by the kidney through urine and by the liver via conjugation to bile acids [14,61]. Submillimolar concentrations of taurine are observed in the plasma (Figure 2A), while much larger concentrations occur in organs with high energy metabolism rates, such as the heart [62,63,64,65].

### 2.1. Brain Taurine Transport

Taurine in the brain results from its transport from the periphery (believed to be the main source) and local de novo synthesis. In most mammals, taurine is mainly synthetized in the liver and then actively transported through the blood-brain barrier into the brain parenchyma. 

Taurine, as well as hypotaurine, β-alanine, and other β-amino acids, are taken up through the blood–brain barrier into the brain by a high-affinity, low-capacity Na^+^- and Cl^−^-dependent transport system [66,67]. The passive diffusion of taurine across the blood-brain barrier is negligible [14]. Taurine uptake or efflux at both luminal and albumen membranes has been proposed to be mediated by SLC6A6 transporter, also called TauT [68]. The blood-brain barrier also expresses the GABA transporter SLC6A13, known as GAT-2, which is capable of carrying taurine across membranes [69,70]. Both TauT and GAT-2 are also able to efficiently carry hypotaurine [71]. Genetic deletion of the taurine transporter (TauT) in mice reduces taurine concentrations in plasma and tissues, including the brain [37]. In contrast, genetic deletion of GAT-2 in mice increases brain taurine levels, suggesting that GAT-2 is mainly functioning as a brain-to-blood efflux system for taurine [69]. 

TauT is expressed in astrocytes and to a lesser extent in neurons [72,73]. GAT-2 expression appears restricted to leptomeninges and blood vessels [74]. Taurine is also transported by ubiquitously expressed volume-sensitive organic osmolyte–anion channels, commonly called volume-regulated anion channels (VRACs), that are activated by cell swelling (see [75] and references therein). Within the brain parenchyma, it has been proposed that taurine uptake is mediated by TauT, while taurine release is mostly mediated by VRACs. Furukawa et al., have shown that that taurine uptake is blocked by a TauT inhibitor and taurine release is blocked by a VRAC blocker in the developing mouse neocortex [76].

### 2.2. Taurine Metabolism

The synthesis of taurine occurs from the catabolism of cysteine in both neurons and astrocytes (Figure 1) and is limited by the oxidation of hypotaurine [77,78]. Cysteine dioxygenase and cysteine sulfinate decarboxylase are concerted to produce hypotaurine from cysteine. Genetic deletion of cysteine dioxygenase in mice depletes hypotaurine and taurine, while causing the accumulation of cysteine and cysteine-containing metabolites such as glutathione [48]. Genetic deletion of cysteine sulfinate decarboxylase also reduces taurine levels in the brain (four-fold less than in controls), as well as in the plasma and other tissues [79]. Either of these mouse models shows impaired development, including reduced brain volume. Cysteamine can also be converted to hypotaurine via cysteamine dioxygenase. The identity of the enzyme that catalyzes the biosynthesis of taurine from hypotaurine, which is denominated hypotaurine dehydrogenase, has remained elusive. Recently, Veeravalli et al., proposed that the oxygenation of hypotaurine to taurine is mainly catalyzed by flavin-containing monooxygenase 1 [80]. Accordingly, the developmental expression of this enzyme in the mouse brain [81] accompanies the developmental decay of brain taurine levels (Figure 2).

Neurons and astrocytes express taurine transporters (e.g., [82]) and release hypotaurine and/or taurine originating from cysteine oxidation [77,78]. However, it remains to be experimentally determined whether taurine metabolism is interdependently regulated by neurons and astrocytes, as proposed elsewhere (see discussion by Banerjee et al. [83]).

### 2.3. Sulphur-Containing Amino Acids

Taurine is not used for protein synthesis. In contrast, the sulphur-containing amino acids methionine and cysteine are protein components and play important roles in maintaining protein structure. While methionine is a very hydrophobic amino acid that contributes to interactions such as those between proteins and lipid bilayers, cysteine mainly participates in protein folding by the formation of disulfide bonds with other cysteine residues [84]. Methionine can be metabolized to the cofactor S-adenosylmethionine that participates in a number of metabolic pathways by acting as a methyl donor, including epigenetic regulation [85] and catecholamine metabolism (epinephrine synthesis) [86]. Such transmethylation reactions can be funneled to produce homocysteine that generates cysteine through transsulfuration [85,87]. Notably, both methionine and cysteine produced from protein degradation can generate taurine as an end-product [88].

## 3. Taurine in Cellular Physiology

### 3.1. Osmoregulation by Taurine

Cells swell and shrink when challenged with osmotic changes. The regulation of cell volume in response to extracellular or intracellular stimuli or osmotic changes is critical for cellular homeostasis. Neuronal activity is associated with changes in cell membrane polarization as a result of active ion fluxes and involves cell volume regulation (e.g., [89]). Pathological edema resulting from cellular swelling occurs in hypo-osmotic conditions or in the presence of cytotoxic ion imbalance. While water is taken up via aquaporin-4 mainly expressed in astrocytes, it has been reported that both neurons and astrocytes swell during acute hypo-osmotic stress (e.g., [90]). As a reaction to cell swelling, several low-molecular-weight organic compounds will influence intracellular osmolarity. 

Taurine occurs in its zwitterionic form over the physiological pH range, turning into an excellent metabolite for osmolarity regulation [14,91]. Indeed, neurons and astrocytes exposed to exogenous taurine up to 10 mmol/L are able to take up extracellular taurine without changes in cell volume [92]. Consistent with a tight regulation of taurine concentration for its action as an organic osmolyte, exposure of brain cells to cysteine or cysteamine results in elevated hypotaurine, but not taurine, levels [78]. Superfused acute mouse cerebral cortical slices regulate taurine release upon osmotic challenges [93]. Brain taurine levels decline over 2 weeks of hyponatremia in rats in vivo [94], while increasing during hypernatremia [95]. Accordingly, taurine synthesis is stimulated under hypertonic conditions in cultured neurons [78]. Astrocytes in a hyperosmotic medium accumulate taurine [96,97]. This is likely due to the increased expression of TauT for taurine uptake rather than to the stimulation of taurine synthesis [98]. In contrast, astrocytes cultured in a hypo-osmotic medium release taurine [99], a process likely mediated by VRAC [100]. While osmotic pressure is regulated by taurine, there are other effects of this compound on the balance of K^+^ and Ca^2+^, which might have implications for neurotransmission [92].

### 3.2. Taurine as a Neurotransmitter

Early work reported taurine uptake into synaptosomes and its release upon electrical stimulation [101,102], as well as taurine binding to synaptosomal membranes [103,104]. Such observations suggested a role of taurine as a neurotransmitter in the central nervous system (CNS); in fact, taurine turned out to be a modulator of inhibitory neurotransmission. 

**Figure 3:**
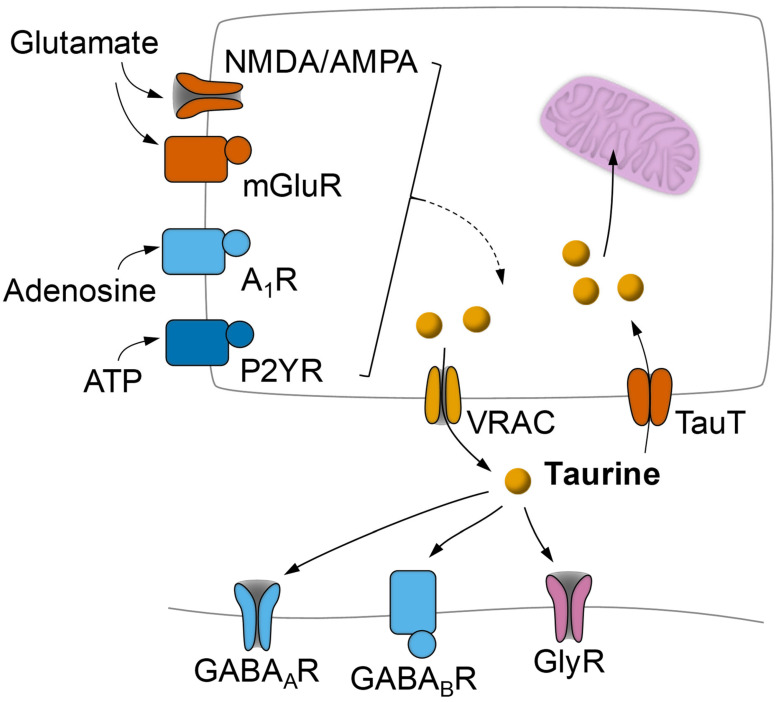
Schematic representation of activity-dependent taurine release modulation from neurons or astrocytes by glutamate and purines and action of taurine on inhibitory receptors. Taurine release is mainly mediated by volume-regulated anion channels (VRAC) that are activated by hypo-osmotic conditions and electrical activity and can be stimulated via glutamate metabotropic (mGluR) and ionotropic receptors (mainly NMDA and AMPA), adenosine A_1_ receptors (A_1_R), and metabotropic ATP receptors (P2Y). Taurine mediates its neuromodulatory effects by binding to GABA_A_, GABA_B_, and glycine receptors. Reuptake of taurine occurs vis the taurine transporter TauT.

γ-Aminobutyric acid (GABA) and glycine are amino acids that mediate inhibitory transmission at chemical synapses. GABAergic synapses employ three types of postsynaptic receptors: the ionotropic GABA_A_ and GABA_C_ that are permeable to Cl^−^ and the metabotropic GABA_B_. Glycine receptors are also permeable to Cl^−^ upon ligand binding. Taurine is known to interact with GABA_A_, GABA_B_, and glycine receptors (Figure 3; [12,105]). While taurine binding to GABA_A_ and GABA_B_ is weaker than to GABA, taurine is a rather potent ligand of the glycine receptor [105].

Intracellular taurine concentration is estimated to be 400-fold higher than the concentration in the extracellular space [30]. Taurine concentration in the brain measured extracellularly using microdialysis is generally below 10 µmol/L, and increases by at least one order of magnitude upon depolarization [106,107,108]. After release, taurine acts on GABA and glycine receptors and is cleared through sodium-dependent transport (see above). Taurine release does not take place exclusively at synapses but can be of glial origin [109,110,111,112] and mediate astrocyte-to-neuron communication [110,113]. 

Concentrations of taurine below 1 mmol/L are rather selective for glycine receptors, as observed in neurons in the basolateral amygdala [114], supraoptic nucleus [115], hippocampus [116], nucleus accumbens [117], and inferior colliculus [118]. Above 1 mmol/L, taurine also activates GABA receptors. However, taurine was shown to act as an endogenous ligand for extra-synaptic GABA_A_ receptors at concentrations ranging from 10 to 100 µmol/L [119]. 

While not modulating glutamatergic neurotransmission, taurine regulates cytoplasmic and intra-mitochondrial Ca^2+^ homeostasis. Therefore, taurine is able to dampen glutamate-induced Ca^2+^ transients in neurons, and thus intracellular Ca^2+^-dependent signaling mediators, and even prevent glutamate excitotoxicity [120,121,122]. Therefore, inhibitory actions of taurine on neuronal excitability might be attributed to a direct enhancement of GABAergic and glycinergic neurotransmission, as well as to the dampening glutamatergic neurotransmission via intracellular effects (discussed by El Idrissi and Trenkner [123]). 

### 3.3. Modulation of Taurine Release in the CNS

In the central nervous system, basal taurine release is largely independent of Ca^2+^, and a Ca^2+^-dependent component can be stimulated by glutamate and K^+^ [124,125,126]. The facilitation of glutamate-induced taurine release is slow and prolonged, varies across the life span, and is mediated by NMDA and AMPA receptors, as well as by kainate receptors in the developing brain [125]. Metabotropic glutamate receptors have also been proposed to modulate taurine release from acute hippocampal slices [127]. Adenosine has been proposed to modulate both basal and K^+^-stimulated taurine release from mouse hippocampal slices via A_1_ receptors [126]. While the activation of adenosine A_1_ receptors enhanced the basal taurine release and stimulated it in hippocampal slices from the developing mouse, it inhibited the basal but not the stimulated release in adults. Purinergic activation by ATP was also proposed to stimulate taurine efflux in cultured rat hippocampal neurons [128]. ATP caused a dose-dependent loss of taurine mediated by P2Y rather than P2X receptors, which could be blocked by a VRAC inhibitor. In sum, taurine release appears to be physiologically regulated by glutamatergic activity and their modulators (Figure 3), namely, purines. 

### 3.4. Taurine in Mitochondria

Taurine concentrations in the brain mitochondria are in the same order of magnitude than those found in other subcellular compartments, such as synaptosomes [129]. Recently, in cultured HeLa cells, taurine concentrations in the mitochondrial matrix were also determined to be similar to those in the whole cell [130]. The authors further found that blocking the complex I with piericidin reduced taurine levels by 40%, but no substantial effects on taurine concentrations in the matrix were found when inhibiting complex II or ATP synthase [130]. 

Taurine amino group with a pKa of 8.6 at 37 °C is suitable for acting as a mitochondrial matrix pH buffer [131]. The regulation of mitochondrial pH is important for brain function, since mitochondrial metabolism in both neurons and astrocytes responds to brain activity (see [132] and references therein). The proton gradient and mitochondrial membrane potential are the drivers of the proton-motive force that produces ATP. Like other cells, neurons and astrocytes in culture show a mitochondrial matrix pH of 7.5–8 [133,134,135]. For example, the uptake of glutamate by astrocytes after synaptic release triggers intracellular acidification that spreads over the mitochondrial matrix [134]. The authors further showed that glutamate-induced mitochondrial matrix acidification exceeded cytosolic acidification and dissipated the cytosol-to-mitochondrial matrix pH gradient, which resulted in the modulation of metabolism and oxygen consumption [131,134,136]. On the other hand, the pH in the mitochondrial matrix of neurons increased upon exposure to excitotoxic levels of glutamate [133]. Taurine might counteract extreme mitochondrial pH fluctuations and help preserve mitochondrial physiology. Mohammadi et al., exposed mitochondria isolated from the mouse liver to a wide range of exogenous taurine concentrations and found that taurine participates in regulating mitochondrial potential, Ca^2+^-induced mitochondrial swelling, the activity of mitochondrial dehydrogenases, and ATP concentration [137]. Mitochondria isolated from the mouse brain or liver show inhibited mitochondrial dehydrogenases activity, collapse of mitochondrial membrane potential, induced mitochondrial swelling, and increased levels of reactive oxygen species upon exposure to ammonia, which are all mitigated by taurine [138].

Taurine is not able to act as a radical scavenger [139]. However, beneficial antioxidant effects of taurine in cells have mostly been linked to improved mitochondrial action and reduced generation of mitochondrial superoxide. Taurine administration to isolated mitochondria from liver or brain was shown to mitigate ammonia-induced mitochondrial dysfunction, including preventing or ameliorating the ammonia-induced collapse of mitochondrial membrane potential, mitochondrial swelling, ATP depletion, and increased reactive oxygen species and oxidative stress [138]. Taurine also decreased the activity of glutathione peroxidase and manganese-superoxide dismutase upon tamoxifen toxicity, which contributed to decreasing mitochondrial oxidative stress, measured through lipid peroxidation, protein carbonyl content, and superoxide radical generation [140]. 

Taurine is a component of mitochondrial tRNAs in taurine-containing modified uridines that are indispensable for protein translation [141,142]. This taurine modification is catalyzed by the enzyme mitochondrial optimization-1, whose deficiency impairs mitochondrial protein translation and ultimately the efficiency of respiration [143]. Several diseases have been directly associated with the lack taurine modification of mitochondrial tRNA [144,145]. 

In sum, taurine supplementation is proposed to improve the function of the mitochondria, contributing to the preservation of mitochondrial membrane potential, proton gradient, and matrix pH that are critical for energy metabolism and efficient oxidative phosphorylation, as well as intracellular calcium homeostasis. 

### 3.5. Taurine as an Inhibitor of Apoptosis

Taurine was found to prevent apoptosis upon many noxious challenges (e.g., [146,147,148]). The most striking neuroprotective effects of taurine were observed on the reduction of apoptotic rates and the improvement of neurological outcomes upon brain ischemia. The suggested mechanisms include the prevention of mitochondrial and endoplasmic reticulum (ER) stress. Taurine was found to attenuate mitochondria-dependent cell death in the ischemic core and penumbra of stroke models by stimulating the antioxidant machinery, preventing energy charge dampening, inhibiting the reduction of anti-apoptotic Bcl-xL and the increase of the pro-apoptotic Bax, preventing cytochrome C release from the mitochondria, and inhibiting the activation of calpain and caspase-3 [149,150,151]. Taurine was also found to prevent ischemia/hypoxia-induced endoplasmic reticulum (ER) stress by inhibiting the unfolded protein response via transcription factor 6 (ATF6), protein kinase R-like ER kinase (PERK), and inositol-requiring enzyme 1 (IRE1) pathways [152,153]. 

## 4. Brain Taurine in Diabetes

Diabetes and many factors of the metabolic syndrome impact the brain, leading to metabolic alterations, synaptic dysfunction, gliosis, and memory impairment [154,155]. MRS studies on rats rendered diabetic by streptozotocin administration showed increased taurine concentrations in the hippocampus (+23%) [156] and cortex (+8%) [157], which is consistent with increased brain taurine uptake in this model [31]. Non-obese, insulin resistant Goto-Kakizaki rats also display increased taurine concentration in the hippocampus (+22%), a brain area involved in learning and memory, relative to Wistar control rats [158]. Brain taurine alterations have also been reported in diet-induced obesity models. Namely, mice fed a lard-based 60%-fat-rich diet for 6 months showed increased taurine in the cortex (+7%), hypothalamus (+9%), and, most prominently, hippocampus (+12%), when compared to low-fat-fed mice [159]. Recently, we further demonstrated that a high-fat and high-sugar diet led to increased hippocampal levels of taurine after 4 weeks, which persisted for several months (ranging from +8% to +14% relative to low-fat-diet-fed controls), which were reversed by diet normalization [38]. Such increase in brain taurine levels in mice with diabetes might have resulted from a compensatory mechanism for cellular protection against metabolic syndrome. 

While increased hippocampal taurine concentrations have been reported in the brain of diabetes models, that remains to be demonstrated in individuals with diabetes (reviewed and discussed in [160]). The lack of evidence on alterations of brain taurine levels in diabetes patients is inherent to the relatively low levels of taurine in the human brain (see Figure 2), and to the difficulty in distinguishing taurine peaks at the weak magnetic fields used in clinical MRS studies (discussed in [161]). However, MRS at higher magnetic fields, namely, at 7 T and above, improves the ability to examine taurine in the living human brain. While not many MRS studies on diabetes individuals are available, other neurodegenerative disorders have been more studied, including Alzheimer’s disease (AD).

### 4.1. Brain Taurine Levels in Subjects with Alzheimer’s Disease

There is a growing body of epidemiological evidence suggesting that obesity and insulin resistance increases the risk of developing age-related cognitive decline, mild cognitive impairment, vascular dementia, and AD, and molecular and metabolic mechanisms linking T2D and AD have been proposed [154,162,163]. While there are limited studies measuring brain taurine in patients with diabetes, research from the AD field might provide additional clues on taurine alterations upon neurodegeneration.

Little attention has been given to taurine concentrations measured by MRS in the brain of AD patients relative to those in healthy individuals ([164,165] and references therein). That is because most MRS studies were conducted at low magnetic fields. In a recent MRS study conducted at 7.0 T, Marjańska et al., found similar concentrations of taurine in AD individuals and age- and gender-matched cognitively healthy controls in the posterior cingulate cortex, a region known to be impacted by AD, and the occipital cortex [166]. Early studies on AD patients also found no substantial changes in cerebrospinal fluid (CSF) taurine levels [167,168] or post-mortem brain taurine levels [169,170]. These studies, however, might be biased by confounding effects from previous medications. Indeed, taurine levels were found reduced (up to −36%) in the CSF of individuals diagnosed with dementia and probable AD who had never been treated with antidepressant or neuroleptic medications [171] and in individuals with advanced symptoms of AD [172]. In another study, CSF taurine levels in AD patients correlated significantly with cognitive scores [168]. Altogether, one might speculate that taurine loss in patients with AD is linked to worsened cognitive deterioration.

### 4.2. Plasma Taurine Levels in Individuals with Dementia and Alzheimer’s Disease

Reduced levels of blood taurine (−23% to −40%) have been observed in subjects with Alzheimer’s disease relative to subjects without neurodegenerative symptoms [173]. In another study, low taurine levels were associated with dementia risk but not with AD risk [174]. Therefore, the authors postulated that a low concentration of taurine might be linked to vascular dysfunction (possibly, vascular dementia) rather than to neurodegeneration. Accordingly, low levels of dietary taurine have been linked to hypertension [175], taurine supplementation in a mouse study was implicated in blood flow regulation [176], and a chronic taurine supplementation showed antihypertensive effects in a clinical trial [2]. However, not all studies associate low taurine levels to AD, and higher taurine levels in the plasma have actually been found in patients with mild cognitive impairment (+43%) and Alzheimer’s disease (AD) (+49%) compared to control subjects [177].

### 4.3. Brain Taurine Levels in AD Models 

The transgenic rat model of AD TgF344-AD rat has been reported to develop age-dependent MRS alterations in brain metabolites, including increased taurine levels in the cortex (+35%) at 18 months of age, but not earlier [178]. Age-dependent increased taurine levels were also observed in the hippocampus (+16% to +21%) and cortex (+25%) of McGill-R-Thy1-APP rats, relative to controls [179]. One study on aged transgenic mice carrying the human Swedish APP mutant Tg2576 showed elevated taurine levels in the cortex (+21%) [180]. However, taurine levels were found unaltered during aging in the brain in many other studies on transgenic mouse models of AD (Refs. [181,182,183] and references therein). Altogether, we conclude that the current evidence points towards contrasting findings on brain taurine levels in AD patients and animal models of the disease. 

## 5. Neuroprotection by Taurine 

Neuroprotection by taurine has been reported for many models of brain injury and neurodegeneration. In animal models, taurine treatments have been reported to significantly improve functional recovery after traumatic brain injury [184,185] or ischemic stroke [149,176,186]. Not only taurine has beneficial effects against neurodegeneration, but also it can modulate inflammatory processes. Namely, it has been established that taurine dampens neuroinflammation in animal models of ischemic stroke and traumatic brain injury that develop severe gliosis (e.g., [11,184,186]).

Given its role as an inhibitory transmitter, taurine was shown to reduce seizures in a mouse model of kainite-induced epilepsy and prevent cell death in the hippocampus, as well as microgliosis and astrogliosis [187]. Furthermore, taurine was suggested to protect dopaminergic neurons in a mouse and rat models of Parkinson’s disease, namely, by inhibiting neuroinflammation and microgliosis [188,189]. Taurine was found to ameliorate cellular and neurochemical alterations in the hippocampus of rodents exposed to chronic stress induced by repeated immobilization or noise exposure, with substantial improvements on memory performance [190,191]. Taurine supplementation was also suggested to afford neuroprotection and anti-apoptotic activity, as well as to reduce microglia activation, in a rat model of chronic inflammation induced by the repeated administration of lipopolysaccharide that mimics a bacterial infection [192]. 

In aging mice, taurine administration was reported to stimulate hippocampal neurogenesis by increasing the rate of progenitor cell formation and to induce a shift in microglia from activated to resting states [193].

Taurine has been shown to protect neurons against excitotoxicity induced by amyloid-β or glutamate in vitro [121,194]. Moreover, taurine supplementation was reported to recover spatial memory in the APP/PS1 mouse model [195] and to improve glutamatergic activity in the brain of the 5xFAD mouse model [196]. While in both models taurine failed to reduce the rate of amyloid-β deposition, taurine was reported to have the ability to decrease amyloid-β aggregation, while favoring the formation for tau protein fibrils [197].

### 5.1. Taurine Affords Neuroprotection in Diabetes Models 

In streptozotocin-induced diabetic rats (insulin-deficient diabetes), treatment with taurine at a dose of 100 mg/kg i.p. during a month reduced oxidative stress, DNA damage, and inflammatory cytokine levels in the frontal cortex and hippocampus, contributing to improving memory performance [198,199]. A study by Agca et al. [200] demonstrated that a 2% (*w*/*v*) taurine supplementation in drinking water for 8 weeks administered to streptozotocin-treated rats ameliorated the diabetes-induced increase of the transcription factor NF-κβ, involved in inflammatory processes, and the diabetes-induced reduction of Nrf2 and glucose transporters Glut1 and Glut3 in the brain. Rahmeier et al. [201] further showed anti-apoptotic effects of taurine administration (100 mg/kg daily i.p.) in the brain of streptozotocin-treated rats. Li et al. [202] described taurine as a protector against myelin damage of the sciatic nerve in streptozotocin-treated rats through the inhibition of apoptosis of Schwann cells. In mice fed a fat-rich diet, which develop metabolic syndrome, we recently demonstrated that 3% (*w*/*v*) taurine supplemented in the drinking water for 2 months prevented memory impairment [203]. Furthermore, magnetic resonance spectroscopy (MRS) for metabolic profiling in vivo showed that taurine treatment prevented the obesity-induced reduction of the neuronal marker *N*-acetylaspartate in the hippocampus [203]. Energy metabolism impairments were also observed in the hippocampus of high-fat-diet-fed mice in this study but could not be prevented by taurine. However, treatment with *N*-acetylcysteine, which acts as a cysteine donor for the synthesis of taurine as well as glutathione, fully prevented obesity-induced metabolic alterations in the hippocampus. Interestingly, it has also been proposed that taurine treatment increases brain insulin receptor density, in particular in the hippocampus [204], which could improve brain insulin sensitivity and thus have beneficial effects to counteract cognitive impairment [154,162,163]. Altogether, the available literature supports taurine administration as a way of preventing neuronal dysfunction in patients with obesity and diabetes.

### 5.2. Taurine Effectiveness in Diabetes Management 

Taurine supplementation has shown beneficial effects on metabolic syndrome factors in both preclinical and clinical studies. We recently reported a taurine-induced improvement of glucose tolerance in female mice fed a high-fat diet during 2 months, compared to non-taurine-supplemented obese mice [202]. Similar results were described by Ribeiro et al. [205], who used 5% (*w*/*v*) taurine in drinking water for 6 months.

The plasma levels of taurine were found to be slightly lower in individuals with T2D than in healthy subjects [20,21]. Interestingly, plasma taurine was found to inversely correlate with fasting glycemia but not with glycated hemoglobin HbA1_c_ levels [206] and to be independent of obesity or body mass index [20,22]. This suggests that taurine is involved in acute metabolic regulation and glucose homeostasis, but not in the etiology of diabetes. Indeed, plasma taurine is reduced during an euglycemic hyperinsulinemic clamp in healthy individuals [23] or during the metabolic response to exercise [207]. According to the roles of taurine in metabolic regulation, we previously observed that taurine concentration in the hippocampus of streptozotocin-treated diabetic rats could be reduced by acute glycemic normalization by means of insulin administration [156].

Given the lower levels of circulating taurine in subjects with diabetes, it has been speculated that dietary taurine supplementation might contribute to diabetes management. Accordingly, several studies on animal models of diabetes have indicated that taurine supplementation lowers glycaemia and improves insulin secretion and sensitivity (e.g., [205,208,209,210,211,212]). Interestingly, it has been proposed that such effects could also be associated with taurine conjugation to bile acids, such as the formation of tauro–ursodeoxycholic acid [213].

Evidence from studies in humans remains controversial, and taurine supplementation has little or no effect on improving metabolic syndrome or T2D and its complications (reviewed in [214]). The source of controversy regarding taurine effects on diabetes might be the poor study design and the low number of subjects tested. For example, a sufficiently powered, double-blinded, randomized, crossover study, based on the administration of a daily taurine supplementation for 8 weeks found no effect on insulin secretion and action and on plasma lipid levels in overweight men with a positive history of T2D [215]. Nevertheless, the beneficial effects of taurine might contribute to protect the various bodily systems from diabetes complications.

## 6. Conclusions

Overfeeding and sedentary lifestyles drive the development of a systemic metabolic imbalance and the emergence of obesity and prediabetes that are strongly associated with all-cause dementia, Alzheimer’s disease (AD), and vascular dementia (e.g., [216]). Obesity is associated with comorbidities such as hypertension, cardiovascular disease, metabolic syndrome, and insulin resistance or type 2 diabetes [216,217], which might modulate the genetic susceptibility to neurodegenerative disorders [218] and thus constitute a risk factor for cognitive decline [219,220]. The reported cytoprotective actions of taurine contribute to brain health improvements in subjects with obesity and diabetes through various mechanisms that improve neuronal function, such as the modulation of inhibitory neurotransmission and, therefore, the promotion of an excitatory–inhibitory balance, the stimulation of antioxidant systems, and the stabilization of mitochondria and thus of energy production and Ca^2+^ homeostasis. Taurine supplementation in experimental models of obesity and diabetes provides evidence for its effects in the prevention of metabolic syndrome-associated memory dysfunction, but the exact mechanisms of taurine action remain to be ascertained; this should be addressed in future studies. Based on this literature survey, we conclude that further research is indeed necessary for a clear understanding of taurine homeostasis in metabolic disorders with an impact on brain function.

In addition to taurine, the amino acids methionine and cysteine from which taurine can be produced (see Section 2.3) have been associated with obesity and metabolic syndrome [207,221,222], and the modulation of the bioavailability of sulphur-containing amino acids might provide further benefits, e.g., by stimulating the synthesis of the antioxidant glutathione (discussed in [203]).

## Data Availability

Not applicable.

## Abbreviations

AD	Alzheimer’s disease
AMPA	α-amino-3-hydroxy-5-methyl-4-isoxazole propionic acid
CNS	central nervous system
CSF	cerebrospinal fluid
GAT-2	GABA transporter (SLC6A13)
MRS	Magnetic resonance spectroscopy
NMDA	*N*-methyl-D-aspartate
TauT	taurine transporter (SLC6A6)
VRAC	volume-regulated anion channel

## References

[1] Russell D.W. (2003). The Enzymes, Regulation, and Genetics of Bile Acid Synthesis. Annu. Rev. Biochem..

[2] Sun Q., Wang B., Li Y., Sun F., Li P., Xia W., Zhou X., Li Q., Wang X., Chen J. (2016). Taurine Supplementation Lowers Blood Pressure and Improves Vascular Function in Prehypertension. Hypertension.

[3] Azuma J. (1994). Heart Failure Research with Taurine Group Long-Term Effect of Taurine in Congestive Heart Failure: Preliminary Report. Adv. Exp. Med. Biol..

[4] Milei J., Ferreira R., Llesuy S.F., Forcada P., Covarrubias J., Boveris A. (1992). Reduction of reperfusion injury with preoperative rapid intravenous infusion of taurine during myocardial revascularization. Am. Hear. J..

[5] Zhao H., Qu J., Li Q., Cui M., Wang J., Zhang K., Liu X., Feng H., Chen Y. (2017). Taurine supplementation reduces neuroinflammation and protects against white matter injury after intracerebral hemorrhage in rats. Amino Acids.

[6] Giri S.N., Wang Q. (1992). Taurine and Niacin Offer a Novel Therapeutic Modality in Prevention of Chemically-Induced Pulmonary Fibrosis in Hamsters. Adv. Exp. Med. Biol..

[7] De Carvalho F.G., Brandao C.F.C., Muñoz V.R., Batitucci G., Tavares M.E.D.A., Teixeira G.R., Pauli J.R., De Moura L.P., Ropelle E.R., Cintra D.E. (2021). Taurine supplementation in conjunction with exercise modulated cytokines and improved subcutaneous white adipose tissue plasticity in obese women. Amino Acids.

[8] Jakaria M., Azam S., Haque M.E., Jo S.-H., Uddin M.S., Kim I.-S., Choi D.-K. (2019). Taurine and its analogs in neurological disorders: Focus on therapeutic potential and molecular mechanisms. Redox Biol..

[9] Yeon J.-A., Kim S.-J. (2010). Neuroprotective Effect of Taurine against Oxidative Stress-Induced Damages in Neuronal Cells. Biomol. Ther..

[10] Rezaee-Tazangi F., Zeidooni L., Rafiee Z., Fakhredini F., Kalantari H., Alidadi H., Khorsandi L. (2020). Taurine effects on Bisphenol A-induced oxidative stress in the mouse testicular mitochondria and sperm motility. JBRA Assist. Reprod..

[11] Nakajima Y., Osuka K., Seki Y., Gupta R.C., Hara M., Takayasu M., Wakabayashi T. (2010). Taurine Reduces Inflammatory Responses after Spinal Cord Injury. J. Neurotrauma.

[12] Albrecht J., Schousboe A. (2005). Taurine Interaction with Neurotransmitter Receptors in the CNS: An Update. Neurochem. Res..

[13] Oja S.S., Saransaari P. (2017). Significance of Taurine in the Brain. Adv. Exp. Med. Biol..

[14] Huxtable R.J. (1992). Physiological actions of taurine. Physiol. Rev..

[15] Jong C.J., Sandal P., Schaffer S.W. (2021). The Role of Taurine in Mitochondria Health: More Than Just an Antioxidant. Molecules.

[16] Wharton B., Morley R., Isaacs E.B., Cole T.J., Lucas A. (2004). Low plasma taurine and later neurodevelopment. Arch. Dis. Child.-Fetal Neonatal Ed..

[17] Sturman J., Moretz R., French J., Wisniewski H. (1985). Taurine deficiency in the developing cat: Persistence of the cerebellar external granule cell layer. J. Neurosci. Res..

[18] Rak K., Völker J., Jürgens L., Scherzad A., Schendzielorz P., Radeloff A., Jablonka S., Mlynski R., Hagen R. (2014). Neurotrophic effects of taurine on spiral ganglion neurons in vitro. NeuroReport.

[19] Mersman B., Zaidi W., Syed N.I., Xu F. (2020). Taurine Promotes Neurite Outgrowth and Synapse Development of Both Vertebrate and Invertebrate Central Neurons. Front. Synaptic Neurosci..

[20] Zhou Y., Qiu L., Xiao Q., Wang Y., Meng X., Xu R., Wang S., Na R. (2013). Obesity and diabetes related plasma amino acid alterations. Clin. Biochem..

[21] De Luca G., Calpona P., Caponetti A., Romano G., Di Benedetto A., Cucinotta D., Di Giorgio R. (2001). Taurine and osmoregulation: Platelet taurine content, uptake, and release in type 2 diabetic patients. Metabolism.

[22] Elshorbagy A.K., Valdivia-Garcia M., Graham I.M., Reis R.P., Luis A.S., Smith A.D., Refsum H. (2012). The association of fasting plasma sulfur-containing compounds with BMI, serum lipids and apolipoproteins. Nutr. Metab. Cardiovasc. Dis..

[23] Tessari P., Kiwanuka E., Coracina A., Zaramella M., Vettore M., Valerio A., Garibotto G. (2005). Insulin in methionine and homocysteine kinetics in healthy humans: Plasma vs. intracellular models. Am. J. Physiol. Metab..

[24] Berson E.L., Schmidt S.Y., Rabin A.R. (1976). Plasma amino-acids in hereditary retinal disease. Ornithine, lysine, and taurine. Br. J. Ophthalmol..

[25] Chiarla C., Giovannini I., Siegel J.H., Boldrini G., Castagneto M. (2000). The Relationship between Plasma Taurine and Other Amino Acid Levels in Human Sepsis. J. Nutr..

[26] Engel J.M., Mühling J., Weiss S., Kärcher B., Lohr T., Menges T., Little S., Hempelmann G. (2005). Relationship of taurine and other amino acids in plasma and in neutrophils of septic trauma patients. Amino Acids.

[27] Rana S.K., Sanders T.A.B. (1986). Taurine concentrations in the diet, plasma, urine and breast milk of vegans compared with omnivores. Br. J. Nutr..

[28] Suleiman M.-S., Rodrigo G.C., Chapman R. (1992). Interdependence of intracellular taurine and sodium in guinea pig heart. Cardiovasc. Res..

[29] Schønheyder F., Lyngbye J. (1962). Influence of partial starvation and of acute scurvy on the free amino acids in blood plasma and muscle in the guinea-pig. Br. J. Nutr..

[30] Lerma J., Herranz A., Herreras O., Abraira V., del Rio R.M. (1986). In vivo determination of extracellular concentration of amino acids in the rat hippocampus. A method based on brain dialysis and computerized analysis. Brain Res..

[31] Trachtman H., Futterweit S., Sturman J.A. (1992). Cerebral Taurine Transport Is Increased During Streptozocin-Induced Diabetes in Rats. Diabetes.

[32] Brand H.S., Chamuleau R.A.F.M., Jörning G.G. (1998). Changes in urinary taurine and hypotaurine excretion after two-thirds hepatectomy in the rat. Amino Acids.

[33] Larsen L.H., Ørstrup L.K.H., Hansen S.H., Grunnet N., Quistorff B., Mortensen O.H. (2015). Fructose Feeding Changes Taurine Homeostasis in Wistar Rats. Adv. Exp. Med. Biol..

[34] Cardoso S., Carvalho C., Santos R., Correia S., Santos M.S., Seiça R., Oliveira C.R., Moreira P.I. (2010). Impact of STZ-induced hyperglycemia and insulin-induced hypoglycemia in plasma amino acids and cortical synaptosomal neurotransmitters. Synapse.

[35] Ma Y., Maruta H., Sun B., Wang C., Isono C., Yamashita H. (2021). Effects of long-term taurine supplementation on age-related changes in skeletal muscle function of Sprague–Dawley rats. Amino Acids.

[36] Chesney R.W., Jax D.K. (1979). Developmental Aspects of Renal beta-Amino Acid Transport, I. Ontogeny of Taurine Reabsorption and Accumulation in Rat Renal Cortex. Pediatr. Res..

[37] Warskulat U., Borsch E., Reinehr R., Heller-Stilb B., Mönnighoff I., Buchczyk D., Donner M., Flögel U., Kappert G., Soboll S. (2006). Chronic liver disease is triggered by taurine transporter knockout in the mouse. FASEB J..

[38] Garcia-Serrano A.M., Mohr A.A., Philippe J., Skoug C., Spégel P., Duarte J.M.N. (2022). Cognitive Impairment and Metabolite Profile Alterations in the Hippocampus and Cortex of Male and Female Mice Exposed to a Fat and Sugar-Rich Diet are Normalized by Diet Reversal. Aging Dis..

[39] Tao Y., He M., Yang Q., Ma Z., Qu Y., Chen W., Peng G., Teng D. (2019). Systemic taurine treatment provides neuroprotection against retinal photoreceptor degeneration and visual function impairments. Drug Des. Dev. Ther..

[40] Taranukhin A.G., Taranukhina E.Y., Saransaari P., Podkletnova I.M., Pelto-Huikko M., Oja S.S. (2010). Neuroprotection by taurine in ethanol-induced apoptosis in the developing cerebellum. J. Biomed. Sci..

[41] Hadj-Saïd W., Froger N., Ivkovic I., Jiménez-López M., Dubus É., Dégardin-Chicaud J., Simonutti M., Quénol C., Neveux N., Villegas-Pérez M.P. (2016). Quantitative and Topographical Analysis of the Losses of Cone Photoreceptors and Retinal Ganglion Cells Under Taurine Depletion. Investig. Opthalmol. Vis. Sci..

[42] Gambarota G., Mekle R., Xin L., Hergt M., Van Der Zwaag W., Krueger G., Gruetter R. (2008). In vivo measurement of glycine with short echo-time 1H MRS in human brain at 7 T. Magn. Reson. Mater. Phys. Biol. Med..

[43] Mekle R., Mlynarik V., Gambarota G., Hergt M., Krueger G., Gruetter R. (2009). MR spectroscopy of the human brain with enhanced signal intensity at ultrashort echo times on a clinical platform at 3T and 7T. Magn. Reson. Med..

[44] Deelchand D.K., Van de Moortele P.-F., Adriany G., Iltis I., Andersen P., Strupp J.P., Vaughan J.T., Uğurbil K., Henry P.-G. (2010). In vivo1H NMR spectroscopy of the human brain at 9.4T: Initial results. J. Magn. Reson..

[45] Schaller B., Mekle R., Xin L., Kunz N., Gruetter R. (2013). Net increase of lactate and glutamate concentration in activated human visual cortex detected with magnetic resonance spectroscopy at 7 tesla. J. Neurosci. Res..

[46] Marjańska M., Auerbach E.J., Valabrègue R., Van de Moortele P.-F., Adriany G., Garwood M. (2011). Localized1H NMR spectroscopy in different regions of human brainin vivoat 7 T:T2relaxation times and concentrations of cerebral metabolites. NMR Biomed..

[47] Marjańska M., McCarten J.R., Hodges J., Hemmy L.S., Grant A., Deelchand D.K., Terpstra M. (2017). Region-specific aging of the human brain as evidenced by neurochemical profiles measured noninvasively in the posterior cingulate cortex and the occipital lobe using 1 H magnetic resonance spectroscopy at 7 T. Neuroscience.

[48] Ueki I., Roman H.B., Valli A., Fieselmann K., Lam J., Peters R., Hirschberger L.L., Stipanuk M.H. (2011). Knockout of the murine cysteine dioxygenase gene results in severe impairment in ability to synthesize taurine and an increased catabolism of cysteine to hydrogen sulfide. Am. J. Physiol. Metab..

[49] Wang W.-T., Lee P., Dong Y., Yeh H.-W., Kim J., Weiner C.P., Brooks W.M., Choi I.-Y. (2016). In Vivo Neurochemical Characterization of Developing Guinea Pigs and the Effect of Chronic Fetal Hypoxia. Neurochem. Res..

[50] Lei H., Berthet C., Hirt L., Gruetter R. (2009). Evolution of the Neurochemical Profile after Transient Focal Cerebral Ischemia in the Mouse Brain. J. Cereb. Blood Flow Metab..

[51] Lei H., Duarte J.M., Mlynarik V., Python A., Gruetter R. (2009). Deep thiopental anesthesia alters steady-state glucose homeostasis but not the neurochemical profile of rat cortex. J. Neurosci. Res..

[52] Xin L., Gambarota G., Duarte J.M.N., Mlynárik V., Gruetter R. (2010). Direct in vivo measurement of glycine and the neurochemical profile in the rat medulla oblongata. NMR Biomed..

[53] Harris J.L., Yeh H.-W., Swerdlow R.H., Choi I.-Y., Lee P., Brooks W.M. (2014). High-field proton magnetic resonance spectroscopy reveals metabolic effects of normal brain aging. Neurobiol. Aging.

[54] Sonnay S., Duarte J.M., Just N., Gruetter R. (2016). Compartmentalised energy metabolism supporting glutamatergic neurotransmission in response to increased activity in the rat cerebral cortex: A 13C MRS study in vivo at 14.1 T. J. Cereb. Blood Flow Metab..

[55] Cuellar-Baena S., Landeck N., Sonnay S., Buck K., Mlynarik V., Zandt R.I., Kirik D. (2016). Assessment of brain metabolite correlates of adeno-associated virus-mediated over-expression of human alpha-synuclein in cortical neurons by in vivo 1 H-MR spectroscopy at 9.4 T. J. Neurochem..

[56] Kulak A., Duarte J.M.N., Do K.Q., Gruetter R. (2010). Neurochemical profile of the developing mouse cortex determined by in vivo1H NMR spectroscopy at 14.1 T and the effect of recurrent anaesthesia. J. Neurochem..

[57] Das Neves Duarte J.M., Kulak A., Gholam-Razaee M.M., Cuenod M., Gruetter R., Do K.Q. (2012). N-Acetylcysteine Normalizes Neurochemical Changes in the Glutathione-Deficient Schizophrenia Mouse Model During Development. Biol. Psychiatry.

[58] Duarte J.M., Do K.Q., Gruetter R. (2014). Longitudinal neurochemical modifications in the aging mouse brain measured in vivo by 1H magnetic resonance spectroscopy. Neurobiol. Aging.

[59] Corcoba A., Steullet P., Duarte J., van de Looij Y., Monin A., Cuenod M., Gruetter R., Do K.Q. (2016). Glutathione Deficit Affects the Integrity and Function of the Fimbria/Fornix and Anterior Commissure in Mice: Relevance for Schizophrenia. Int. J. Neuropsychopharmacol..

[60] Gapp K., Corcoba A., Van Steenwyk G., Mansuy I.M., Duarte J.M. (2016). Brain metabolic alterations in mice subjected to postnatal traumatic stress and in their offspring. J. Cereb. Blood Flow Metab..

[61] Roig-Pérez S., Moretó M., Ferrer R. (2005). Transepithelial Taurine Transport in Caco-2 Cell Monolayers. J. Membr. Biol..

[62] Jacobsen J.G., Smith L.H. (1968). Biochemistry and physiology of taurine and taurine derivatives. Physiol. Rev..

[63] Chesney R.W., Lippincott S., Gusowski N., Padilla M., Zelikovic I. (1986). Studies on Renal Adaptation to Altered Dietary Amino Acid Intake: Tissue Taurine Responses in Nursing and Adult Rats. J. Nutr..

[64] Thaeomor A., Wyss J.M., Jirakulsomchok D., Roysommuti S. (2010). High sugar intake via the renin-angiotensin system blunts the baroreceptor reflex in adult rats that were perinatally depleted of taurine. J. Biomed. Sci..

[65] Wójcik O.P., Koenig K.L., Zeleniuch-Jacquotte A., Costa M., Chen Y. (2010). The potential protective effects of taurine on coronary heart disease. Atherosclerosis.

[66] Rasgado-Flores H., Mokashi A., Hawkins R.A. (2012). Na+-dependent transport of taurine is found only on the abluminal membrane of the blood–brain barrier. Exp. Neurol..

[67] Tamai I., Senmaru M., Terasaki T., Tsuji A. (1995). Na+- and Cl−-Dependent transport of taurine at the blood-brain barrier. Biochem. Pharmacol..

[68] Lee N.-Y., Kang Y.-S. (2004). The brain-to-blood efflux transport of taurine and changes in the blood–brain barrier transport system by tumor necrosis factor-α. Brain Res..

[69] Zhou Y., Holmseth S., Guo C., Hassel B., Höfner G., Huitfeldt H.S., Wanner K., Danbolt N.C. (2012). Deletion of the γ-Aminobutyric Acid Transporter 2 (GAT2 and SLC6A13) Gene in Mice Leads to Changes in Liver and Brain Taurine Contents. J. Biol. Chem..

[70] Geier E.G., Chen E.C., Webb A., Papp A.C., Yee S.W., Sadee W., Giacomini K.M. (2013). Profiling Solute Carrier Transporters in the Human Blood–Brain Barrier. Clin. Pharmacol. Ther..

[71] Nishimura T., Higuchi K., Yoshida Y., Sugita-Fujisawa Y., Kojima K., Sugimoto M., Santo M., Tomi M., Nakashima E. (2018). Hypotaurine Is a Substrate of GABA Transporter Family Members GAT2/Slc6a13 and TAUT/Slc6a. Biol. Pharm. Bull..

[72] Pow D.V., Sullivan R., Reye P., Hermanussen S. (2002). Localization of taurine transporters, taurine, and3H taurine accumulation in the rat retina, pituitary, and brain. Glia.

[73] Fujita T., Shimada A., Wada M., Miyakawa S., Yamamoto A. (2006). Functional Expression of Taurine Transporter and its Up-Regulation in Developing Neurons from Mouse Cerebral Cortex. Pharm. Res..

[74] Durkin M.M., Smith K.E., Borden L.A., Weinshank R.L., Branchek T.A., Gustafson E.L. (1995). Localization of messenger RNAs encoding three GABA transporters in rat brain: An in situ hybridization study. Mol. Brain Res..

[75] Mongin A.A. (2016). Volume-regulated anion channel—A frenemy within the brain. Pflügers Arch. Eur. J. Physiol..

[76] Furukawa T., Yamada J., Akita T., Matsushima Y., Yanagawa Y., Fukuda A. (2014). Roles of taurine-mediated tonic GABAA receptor activation in the radial migration of neurons in the fetal mouse cerebral cortex. Front. Cell. Neurosci..

[77] Brand A., Leibfritz D., Hamprecht B., Dringen R. (2002). Metabolism of Cysteine in Astroglial Cells: Synthesis of Hypotaurine and Taurine. J. Neurochem..

[78] Vitvitsky V., Garg S.K., Banerjee R. (2011). Taurine Biosynthesis by Neurons and Astrocytes. J. Biol. Chem..

[79] Park E., Park S.Y., Dobkin C., Schuller-Levis G. (2014). Development of a Novel Cysteine Sulfinic Acid Decarboxylase Knockout Mouse: Dietary Taurine Reduces Neonatal Mortality. J. Amino Acids.

[80] Veeravalli S., Phillips I.R., Freire R.T., Varshavi D., Everett J.R., Shephard E.A. (2020). Flavin-Containing Monooxygenase 1 Catalyzes the Production of Taurine from Hypotaurine. Drug Metab. Dispos..

[81] Janmohamed A., Hernandez D., Phillips I.R., Shephard E. (2004). Cell-, tissue-, sex- and developmental stage-specific expression of mouse flavin-containing monooxygenases (Fmos). Biochem. Pharmacol..

[82] Junyent F., De Lemos L., Utrera J., Paco S., Aguado F., Camins A., Pallàs M., Romero R., Auladell C. (2011). Content and traffic of taurine in hippocampal reactive astrocytes. Hippocampus.

[83] Banerjee R., Vitvitsky V., Garg S.K. (2008). The undertow of sulfur metabolism on glutamatergic neurotransmission. Trends Biochem. Sci..

[84] Brosnan J.T., Brosnan M.E. (2006). The Sulfur-Containing Amino Acids: An Overview. J. Nutr..

[85] Ouyang Y., Wu Q., Li J., Sun S., Sun S. (2020). S-adenosylmethionine: A metabolite critical to the regulation of autophagy. Cell Prolif..

[86] Kvetnansky R., Sabban E.L., Palkovits M. (2009). Catecholaminergic Systems in Stress: Structural and Molecular Genetic Approaches. Physiol. Rev..

[87] Sbodio J.I., Snyder S.H., Paul B.D. (2019). Regulators of the transsulfuration pathway. Br. J. Pharmacol..

[88] Stipanuk M.H., Ueki I. (2011). Dealing with methionine/homocysteine sulfur: Cysteine metabolism to taurine and inorganic sulfur. J. Inherit. Metab. Dis..

[89] Churchwell K.B., Wright S.H., Emma F., Rosenberg P., Strange K. (1996). NMDA Receptor Activation Inhibits Neuronal Volume Regulation after Swelling Induced by Veratridine-Stimulated Na+Influx in Rat Cortical Cultures. J. Neurosci..

[90] Murphy T.R., Davila D., Cuvelier N., Young L.R., Lauderdale K., Binder D.K., Fiacco T.A. (2017). Hippocampal and Cortical Pyramidal Neurons Swell in Parallel with Astrocytes during Acute Hypoosmolar Stress. Front. Cell. Neurosci..

[91] Lambert I.H. (2004). Regulation of the cellular content of the organic osmolyte taurine in mammalian cells. Neurochem. Res..

[92] Walz W., Allen A.F. (1987). Evaluation of the osmoregulatory function of taurine in brain cells. Exp. Brain Res..

[93] Oja S. (1995). Chloride ions, potassium stimulation and release of endogenous taurine from cerebral cortical slices from 3 day old and 3 month old mice. Neurochem. Int..

[94] Verbalis J., Gullans S. (1991). Hyponatremia causes large sustained reductions in brain content of multiple organic osmolytes in rats. Brain Res..

[95] Lien Y.H., Shapiro J., Chan L. (1990). Effects of hypernatremia on organic brain osmoles. J. Clin. Investig..

[96] Olson J.E., Goldfinger M.D. (1990). Amino acid content of rat cerebral astrocytes adapted to hyperosmotic medium in vitro. J. Neurosci. Res..

[97] Sánchez-Olea R., Morán J., Pasantes-Morales H. (1992). Changes in taurine transport evoked by hyperosmolarity in cultured astrocytes. J. Neurosci. Res..

[98] Bitoun M., Tappaz M. (2002). Taurine Down-Regulates Basal and Osmolarity-Induced Gene Expression of Its Transporter, but Not the Gene Expression of Its Biosynthetic Enzymes, in Astrocyte Primary Cultures. J. Neurochem..

[99] Kimelberg H., Goderie S., Higman S., Pang S., Waniewski R. (1990). Swelling-induced release of glutamate, aspartate, and taurine from astrocyte cultures. J. Neurosci..

[100] Qiu Z., Dubin A.E., Mathur J., Tu B., Reddy K., Miraglia L.J., Reinhardt J., Orth A.P., Patapoutian A. (2014). SWELL1, a Plasma Membrane Protein, Is an Essential Component of Volume-Regulated Anion Channel. Cell.

[101] Schmid R., Sieghart W., Karobath M. (1975). Taurine Uptake in Synaptosomal Fractions of Rat Cerebral Cortex. J. Neurochem..

[102] Lähdesmäki P., Pasula M., Oja S.S. (1975). Effect of electrical stimulation and chlorpromazine on the uptake and release of taurine, γ-aminobutyric acid and glutamic acid in mouse brain synaptosomes. J. Neurochem..

[103] Kontro P., Oja S.S. (1983). Sodium-independent taurine binding to brain synaptic membranes. Cell. Mol. Neurobiol..

[104] Huxtable R., Peterson A. (1989). Sodium-dependent and sodium-independent binding of taurine to rat brain synaptosomes. Neurochem. Int..

[105] Lynch J.W. (2004). Molecular Structure and Function of the Glycine Receptor Chloride Channel. Physiol. Rev..

[106] Shibanoki S., Kogure M., Sugahara M., Ishikawa K. (1993). Effect of Systemic Administration of N-Methyl-d-Aspartic Acid on Extracellular Taurine Level Measured by Microdialysis in the Hippocampal CA1 Field and Striatum of Rats. J. Neurochem..

[107] Segovia G., Del Arco A., Mora F. (1997). Endogenous Glutamate Increases Extracellular Concentrations of Dopamine, GABA, and Taurine Through NMDA and AMPA/Kainate Receptors in Striatum of the Freely Moving Rat: A Microdialysis Study. J. Neurochem..

[108] Holopainen I., Kontro P., Oja S.S. (1985). Release of preloaded taurine and hypotaurine from astrocytes in primary culture: Stimulation by calcium-free media. Neurochem. Res..

[109] Shain W.G., Martin D.L. (1984). Activation of beta-adrenergic receptors stimulates taurine release from glial cells. Cell. Mol. Neurobiol..

[110] Philibert R.A., Rogers K.L., Allen A.J., Dutton G.R. (1988). Dose-Dependent, K+-Stimulated Efflux of Endogenous Taurine from Primary Astrocyte Cultures Is Ca2+-Dependent. J. Neurochem..

[111] Philibert R., Rogers K.L., Dutton G.R. (1989). K+-evoked taurine efflux from cerebellar astrocytes: On the roles of Ca2+ and Na+. Neurochem. Research.

[112] Barakat L., Wang D., Bordey A. (2002). Carrier-mediated uptake and release of taurine from Bergmann glia in rat cerebellar slices. J. Physiol..

[113] Choe K., Olson J.E., Bourque C.W. (2012). Taurine Release by Astrocytes Modulates Osmosensitive Glycine Receptor Tone and Excitability in the Adult Supraoptic Nucleus. J. Neurosci..

[114] McCool B., Botting S.K. (2000). Characterization of strychnine-sensitive glycine receptors in acutely isolated adult rat basolateral amygdala neurons. Brain Res..

[115] Hussy N., Brès V., Rochette M., Duvoid A., Alonso G., Dayanithi G., Moos F.C. (2001). Osmoregulation of Vasopressin Secretion via Activation of Neurohypophysial Nerve Terminals Glycine Receptors by Glial Taurine. J. Neurosci..

[116] Wu Z.-Y., Xu T.-L. (2003). Taurine-evoked chloride current and its potentiation by intracellular Ca2+ in immature rat hippocampal CA1 neurons. Amino Acids.

[117] Jiang Z., Krnjević K., Wang F., Ye J.H. (2004). Taurine Activates Strychnine-Sensitive Glycine Receptors in Neurons Freshly Isolated from Nucleus Accumbens of Young Rats. J. Neurophysiol..

[118] Xu H., Zhou K.-Q., Huang Y.-N., Chen L., Xu T.-L. (2004). Taurine activates strychnine-sensitive glycine receptors in neurons of the rat inferior colliculus. Brain Res..

[119] Jiang Z., Yue M., Chandra D., Keramidas A., Goldstein P., Homanics G., Harrison N.L. (2008). Taurine Is a Potent Activator of Extrasynaptic GABAA Receptors in the Thalamus. J. Neurosci..

[120] El Idrissi A., Trenkner E. (1999). Growth Factors and Taurine Protect against Excitotoxicity by Stabilizing Calcium Homeostasis and Energy Metabolism. J. Neurosci..

[121] Louzada P.R., Lima A.C.P., Mendonca-Silva D.L., Noël F., De Mello F.G., Ferreira S.T. (2004). Taurine prevents the neurotoxicity of beta-amyloid and glutamate receptor agonists: Activation of GABA receptors and possible implications for Alzheimer’s disease and other neurological disorders. FASEB J..

[122] Bulley S., Shen W. (2010). Reciprocal regulation between taurine and glutamate response via Ca2+- dependent pathways in retinal third-order neurons. J. Biomed. Sci..

[123] El Idrissi A., Trenkner E. (2004). Taurine as a Modulator of Excitatory and Inhibitory Neurotransmission. Neurochem. Res..

[124] Saransaari P., Oja S. (1991). Excitatory amino acids evoke taurine release from cerebral cortex slices from adult and developing mice. Neuroscience.

[125] Saransaari P. (1997). Taurine release from the developing and ageing hippocampus: Stimulation by agonists of ionotropic glutamate receptors. Mech. Ageing Dev..

[126] Saransaari P., Oja S. (2000). Modulation of the ischemia-induced taurine release by adenosine receptors in the developing and adult mouse hippocampus. Neuroscience.

[127] Saransaari P.P., Oja S.S. (1999). Involvement of metabotropic glutamate receptors in taurine release in the adult and developing mouse hippocampus. Amino Acids.

[128] Li G., Olson J.E. (2008). Purinergic activation of anion conductance and osmolyte efflux in cultured rat hippocampal neurons. Am. J. Physiol. Physiol..

[129] Bonhaus D.W., Lippincott S.E., Huxtable R.J., Sanchez A.P., Scheffner D. (1984). Subcellular Distribution of Neuroactive Amino Acids in Brains of Genetically Epileptic Rats. Epilepsia.

[130] Chen W., Freinkman E., Wang T., Birsoy K., Sabatini D.M. (2016). Absolute Quantification of Matrix Metabolites Reveals the Dynamics of Mitochondrial Metabolism. Cell.

[131] Hansen S.H., Andersen M.L., Cornett C., Gradinaru R., Grunnet N. (2010). A role for taurine in mitochondrial function. J. Biomed. Sci..

[132] Sonnay S., Poirot J., Just N., Clerc A.-C., Gruetter R., Rainer G., Duarte J.M.N. (2017). Astrocytic and neuronal oxidative metabolism are coupled to the rate of glutamate-glutamine cycle in the tree shrew visual cortex. Glia.

[133] Cano-Abad M.F., Di Benedetto G., Magalhães P.J., Filippin L., Pozzan T. (2004). Mitochondrial pH Monitored by a New Engineered Green Fluorescent Protein Mutant. J. Biol. Chem..

[134] Azarias G., Perreten H., Lengacher S., Poburko D., Demaurex N., Magistretti P.J., Chatton J.-Y. (2011). Glutamate Transport Decreases Mitochondrial pH and Modulates Oxidative Metabolism in Astrocytes. J. Neurosci..

[135] Poburko D., Domingo J.S., Demaurex N. (2011). Dynamic Regulation of the Mitochondrial Proton Gradient during Cytosolic Calcium Elevations. J. Biol. Chem..

[136] Thevenet J., De Marchi U., Domingo J.S., Christinat N., Bultot L., Lefebvre G., Sakamoto K., Descombes P., Masoodi M., Wiederkehr A. (2016). Medium-chain fatty acids inhibit mitochondrial metabolism in astrocytes promoting astrocyte-neuron lactate and ketone body shuttle systems. FASEB J..

[137] Mohammadi H., Ommati M.M., Farshad O., Jamshidzadeh A., Nikbakht M.R., Niknahad H., Heidari R. (2019). Taurine and isolated mitochondria: A concentration-response study. Trends Pharm. Sci..

[138] Niknahad H., Jamshidzadeh A., Heidari R., Zarei M., Ommati M.M. (2017). Ammonia-induced mitochondrial dysfunction and energy metabolism disturbances in isolated brain and liver mitochondria, and the effect of taurine administration: Relevance to hepatic encephalopathy treatment. Clin. Exp. Hepatol..

[139] Aruoma O.I., Halliwell B., Hoey B.M., Butler J. (1988). The antioxidant action of taurine, hypotaurine and their metabolic precursors. Biochem. J..

[140] Parvez S., Tabassum H., Banerjee B.D., Raisuddin S. (2008). Taurine Prevents Tamoxifen-Induced Mitochondrial Oxidative Damage in Mice. Basic Clin. Pharmacol. Toxicol..

[141] Suzuki T., Wada T., Saigo K., Watanabe K. (2002). Taurine as a constituent of mitochondrial tRNAs: New insights into the functions of taurine and human mitochondrial diseases. EMBO J..

[142] Yasukawa T., Kirino Y., Ishii N., Holt I., Jacobs H.T., Makifuchi T., Fukuhara N., Ohta S., Suzuki T., Watanabe K. (2005). Wobble modification deficiency in mutant tRNAs in patients with mitochondrial diseases. FEBS Lett..

[143] Fakruddin, Wei F.-Y., Suzuki T., Asano K., Kaieda T., Omori A., Izumi R., Fujimura A., Kaitsuka T., Miyata K. (2018). Defective Mitochondrial tRNA Taurine Modification Activates Global Proteostress and Leads to Mitochondrial Disease. Cell Rep..

[144] Schaffer S.W., Jong C.J., Ito T., Azuma J. (2012). Role of taurine in the pathologies of MELAS and MERRF. Amino Acids.

[145] Ohsawa Y., Hagiwara H., Nishimatsu S.-I., Hirakawa A., Kamimura N., Ohtsubo H., Fukai Y., Murakami T., Koga Y., Goto Y.-I. (2019). Taurine supplementation for prevention of stroke-like episodes in MELAS: A multicentre, open-label, 52-week phase III trial. J. Neurol. Neurosurg. Psychiatry.

[146] Zhang Y., Li D., Li H., Hou D., Hou J. (2016). Taurine Pretreatment Prevents Isoflurane-Induced Cognitive Impairment by Inhibiting ER Stress-Mediated Activation of Apoptosis Pathways in the Hippocampus in Aged Rats. Neurochem. Res..

[147] Li S., Yang L., Zhang Y., Zhang C., Shao J., Liu X., Li Y., Piao F. (2017). Taurine Ameliorates Arsenic-Induced Apoptosis in the Hippocampus of Mice Through Intrinsic Pathway. Adv. Exp. Med. Biol..

[148] Agarwal R., Arfuzir N.N.N., Iezhitsa I., Agarwal P., Sidek S., Ismail N.M. (2018). Taurine protects against retinal and optic nerve damage induced by endothelin-1 in rats via antioxidant effects. Neural Regen. Res..

[149] Sun M., Xu C. (2007). Neuroprotective Mechanism of Taurine due to Up-regulating Calpastatin and Down-regulating Calpain and Caspase-3 during Focal Cerebral Ischemia. Cell. Mol. Neurobiol..

[150] Sun M., Gu Y., Zhao Y., Xu C. (2011). Protective functions of taurine against experimental stroke through depressing mitochondria-mediated cell death in rats. Amino Acids.

[151] Zhu X.-Y., Ma P.-S., Wu W., Zhou R., Hao Y.-J., Niu Y., Sun T., Li Y.-X., Yu J.-Q. (2016). Neuroprotective actions of taurine on hypoxic-ischemic brain damage in neonatal rats. Brain Res. Bull..

[152] Gharibani P.M., Modi J., Pan C., Menzie J., Ma Z., Chen P.-C., Tao R., Prentice H., Wu J.-Y. (2013). The Mechanism of Taurine Protection Against Endoplasmic Reticulum Stress in an Animal Stroke Model of Cerebral Artery Occlusion and Stroke-Related Conditions in Primary Neuronal Cell Culture. Adv. Exp. Med. Biol..

[153] Gharibani P., Modi J., Menzie J., Alexandrescu A., Ma Z., Tao R., Prentice H., Wu J.-Y. (2015). Comparison between single and combined post-treatment with S-Methyl-N,N-diethylthiolcarbamate sulfoxide and taurine following transient focal cerebral ischemia in rat brain. Neuroscience.

[154] Duarte J.M.N. (2014). Metabolic Alterations Associated to Brain Dysfunction in Diabetes. Aging Dis..

[155] Garcia-Serrano A.M., Duarte J.M.N. (2020). Brain Metabolism Alterations in Type 2 Diabetes: What Did We Learn from Diet-Induced Diabetes Models?. Front. Neurosci..

[156] Duarte J.M.N., Carvalho R., Cunha R., Gruetter R. (2009). Caffeine consumption attenuates neurochemical modifications in the hippocampus of streptozotocin-induced diabetic rats. J. Neurochem..

[157] Wang W.-T., Lee P., Yeh H.-W., Smirnova I.V., Choi I.-Y. (2012). Effects of acute and chronic hyperglycemia on the neurochemical profiles in the rat brain with streptozotocin-induced diabetes detected using in vivo1H MR spectroscopy at 9.4 T. J. Neurochem..

[158] Duarte J.M.N., Skoug C., Silva H.B., Carvalho R., Gruetter R., Cunha R. (2019). Impact of Caffeine Consumption on Type 2 Diabetes-Induced Spatial Memory Impairment and Neurochemical Alterations in the Hippocampus. Front. Neurosci..

[159] Lizarbe B., Soares A.F., Larsson S., Duarte J.M.N. (2019). Neurochemical Modifications in the Hippocampus, Cortex and Hypothalamus of Mice Exposed to Long-Term High-Fat Diet. Front. Neurosci..

[160] Duarte J.M.N. (2016). Metabolism in the Diabetic Brain: Neurochemical Profiling by 1H Magnetic Resonance Spectroscopy. Diabetes Metab. Disord..

[161] Duarte J.M.N., Lei H., Mlynárik V., Gruetter R. (2012). The neurochemical profile quantified by in vivo1H NMR spectroscopy. NeuroImage.

[162] De La Monte S.M. (2017). Insulin Resistance and Neurodegeneration: Progress Towards the Development of New Therapeutics for Alzheimer’s Disease. Drugs.

[163] Barone E., Di Domenico F., Perluigi M., Butterfield D.A. (2021). The interplay among oxidative stress, brain insulin resistance and AMPK dysfunction contribute to neurodegeneration in type 2 diabetes and Alzheimer disease. Free Radic. Biol. Med..

[164] Liu H., Zhang D., Lin H., Zhang Q., Zheng L., Zheng Y., Yin X., Li Z., Liang S., Huang S. (2021). Meta-Analysis of Neurochemical Changes Estimated via Magnetic Resonance Spectroscopy in Mild Cognitive Impairment and Alzheimer’s Disease. Front. Aging Neurosci..

[165] Song T., Song X., Zhu C., Patrick R., Skurla M., Santangelo I., Green M., Harper D., Ren B., Forester B.P. (2021). Mitochondrial dysfunction, oxidative stress, neuroinflammation, and metabolic alterations in the progression of Alzheimer’s disease: A meta-analysis of in vivo magnetic resonance spectroscopy studies. Ageing Res. Rev..

[166] Marjańska M., McCarten J.R., Hodges J.S., Hemmy L.S., Terpstra M. (2019). Distinctive Neurochemistry in Alzheimer’s Disease via 7 T In Vivo Magnetic Resonance Spectroscopy. J. Alzheimer’s Dis..

[167] Degrell I., Hellsing K., Nagy E., Niklasson F. (1989). Amino acid concentrations in cerebrospinal fluid in presenile and senile dementia of Alzheimer type and multi-infarct dementia. Arch. Gerontol. Geriatr..

[168] Vermeiren Y., Le Bastard N., Van Hemelrijck A., Drinkenburg W.H., Engelborghs S., De Deyn P.P. (2013). Behavioral correlates of cerebrospinal fluid amino acid and biogenic amine neurotransmitter alterations in dementia. Alzheimer’s Dement..

[169] Mb D.W.E., Beal M.F., Mazurek M.F., Bird E.D., Martin J.B. (1986). A postmortem study of amino acid neurotransmitters in Alzheimer’s disease. Ann. Neurol..

[170] Perry T.L., Yong V.W., Bergeron C., Ba S.H., Jones K. (1987). Amino acids, glutathione, and glutathione transferase activity in the brains of patients with Alzheimer’s disease. Ann. Neurol..

[171] Pomara N., Singh R., Deptula D., Chou J.C., Schwartz M.B., LeWitt P.A. (1992). Glutamate and other CSF amino acids in Alzheimer’s disease. Am. J. Psychiatry.

[172] Csernansky J.G., Bardgett M.E., Sheline Y.I., Morris J.C., Olney J.W. (1996). CSF excitatory amino acids and severity of illness in Alzheimer’s disease. Neurology.

[173] Aquilani R., Costa A., Maestri R., Ramusino M.C., Pierobon A., Dossena M., Solerte S.B., Condino A.M., Torlaschi V., Bini P. (2020). Mini Nutritional Assessment May Identify a Dual Pattern of Perturbed Plasma Amino Acids in Patients with Alzheimer’s Disease: A Window to Metabolic and Physical Rehabilitation?. Nutrients.

[174] Chouraki V., Preis S.R., Yang Q., Beiser A., Li S., Larson M.G., Weinstein G., Wang T.J., Gerszten R.E., Vasan R.S. (2017). Association of amine biomarkers with incident dementia and Alzheimer’s disease in the Framingham Study. Alzheimer’s Dement..

[175] Roysommuti S., Wyss J.M. (2014). Perinatal taurine exposure affects adult arterial pressure control. Amino Acids.

[176] Wang G., Jiang Z.-L., Fan X.-J., Zhang L., Li X., Ke K.-F. (2007). Neuroprotective effect of taurine against focal cerebral ischemia in rats possibly mediated by activation of both GABAA and glycine receptors. Neuropharmacology.

[177] Ravaglia G., Forti P., Maioli F., Bianchi G., Martelli M., Talerico T., Servadei L., Zoli M., Mariani E. (2004). Plasma amino acid concentrations in patients with amnestic mild cognitive impairment or Alzheimer disease. Am. J. Clin. Nutr..

[178] Chaney A.M., Lopez-Picon F.R., Serrière S., Wang R., Bochicchio D., Webb S.D., Vandesquille M., Harte M.K., Georgiadou C., Lawrence C. (2021). Prodromal neuroinflammatory, cholinergic and metabolite dysfunction detected by PET and MRS in the TgF344-AD transgenic rat model of AD: A collaborative multi-modal study. Theranostics.

[179] Nilsen L.H., Melø T.M., Saether O., Witter M.P., Sonnewald U., Sæther O. (2012). Altered neurochemical profile in the McGill-R-Thy1-APP rat model of Alzheimer’s disease: A longitudinalin vivo1H MRS study. J. Neurochem..

[180] Dedeoglu A., Choi J.-K., Cormier K., Kowall N.W., Jenkins B.G. (2004). Magnetic resonance spectroscopic analysis of Alzheimer’s disease mouse brain that express mutant human APP shows altered neurochemical profile. Brain Res..

[181] Mlynarik V., Cacquevel M., Sun-Reimer L., Janssens S., Cudalbu C., Lei H., Schneider B.L., Aebischer P., Gruetter R. (2012). Proton and phosphorus magnetic resonance spectroscopy of a mouse model of Alzheimer’s disease. J. Alzheimer’s Dis..

[182] Forster D., Davies K., Williams S. (2013). Magnetic resonance spectroscopy in vivo of neurochemicals in a transgenic model of Alzheimer’s disease: A longitudinal study of metabolites, relaxation time, and behavioral analysis in TASTPM and wild-type mice. Magn. Reson. Med..

[183] Chaney A., Bauer M., Bochicchio D., Smigova A., Kassiou M., Davies K.E., Williams S.R., Boutin H. (2017). Longitudinal investigation of neuroinflammation and metabolite profiles in the APPswe × PS 1Δe9 transgenic mouse model of Alzheimer’s disease. J. Neurochem..

[184] Su Y., Fan W., Ma Z., Wen X., Wang W., Wu Q., Huang H. (2014). Taurine improves functional and histological outcomes and reduces inflammation in traumatic brain injury. Neuroscience.

[185] Wang Q., Fan W., Cai Y., Wu Q., Mo L., Huang Z., Huang H. (2016). Protective effects of taurine in traumatic brain injury via mitochondria and cerebral blood flow. Amino Acids.

[186] Sun M., Zhao Y., Gu Y., Xu C. (2011). Anti-inflammatory mechanism of taurine against ischemic stroke is related to down-regulation of PARP and NF-ϰB. Amino Acids.

[187] Junyent F., Utrera J., Romero R., Pallàs M., Camins A., Duque D., Auladell C. (2009). Prevention of epilepsy by taurine treatments in mice experimental model. J. Neurosci. Res..

[188] Che Y., Hou L., Sun F., Zhang C., Liu X., Piao F., Zhang D., Li H., Wang Q. (2018). Taurine protects dopaminergic neurons in a mouse Parkinson’s disease model through inhibition of microglial M1 polarization. Cell Death Dis..

[189] Abuirmeileh A.N., Abuhamdah S.M., Ashraf A., Alzoubi K.H. (2021). Protective effect of caffeine and/or taurine on the 6-hydroxydopamine-induced rat model of Parkinson’s disease: Behavioral and neurochemical evidence. Restor. Neurol. Neurosci..

[190] Haider S., Sajid I., Batool Z., Madiha S., Sadir S., Kamil N., Liaquat L., Ahmad S., Tabassum S., Khaliq S. (2020). Supplementation of Taurine Insulates Against Oxidative Stress, Confers Neuroprotection and Attenuates Memory Impairment in Noise Stress Exposed Male Wistar Rats. Neurochem. Res..

[191] Jangra A., Rajput P., Dwivedi D., Lahkar M. (2020). Amelioration of Repeated Restraint Stress-Induced Behavioral Deficits and Hippocampal Anomalies with Taurine Treatment in Mice. Neurochem. Res..

[192] Silva S.P., Zago A.M., Carvalho F.B., Germann L., Colombo G.D.M., Rahmeier F.L., Gutierres J.M., Reschke C.R., Bagatini M.D., Assmann C.E. (2021). Neuroprotective Effect of Taurine against Cell Death, Glial Changes, and Neuronal Loss in the Cerebellum of Rats Exposed to Chronic-Recurrent Neuroinflammation Induced by LPS. J. Immunol. Res..

[193] Gebara E., Udry F., Sultan S., Toni N. (2015). Taurine increases hippocampal neurogenesis in aging mice. Stem Cell Res..

[194] Paula-Lima A.C., De Felice F.G., Brito-Moreira J., Ferreira S.T. (2005). Activation of GABAA receptors by taurine and muscimol blocks the neurotoxicity of beta-amyloid in rat hippocampal and cortical neurons. Neuropharmacology.

[195] Kim H.Y., Kim H.V., Yoon J.H., Kang B.R., Cho S.M., Lee S., Kim J.Y., Kim J.W., Cho Y., Woo J. (2014). Taurine in drinking water recovers learning and memory in the adult APP/PS1 mouse model of Alzheimer’s disease. Sci. Rep..

[196] Oh S.J., Lee H.-J., Jeong Y.J., Nam K.R., Kang K.J., Han S.J., Lee K.C., Lee Y.J., Choi J.Y. (2020). Evaluation of the neuroprotective effect of taurine in Alzheimer’s disease using functional molecular imaging. Sci. Rep..

[197] Santa-María I., Hernández F., Moreno F.J., Avila J. (2007). Taurine, an inducer for tau polymerization and a weak inhibitor for amyloid-beta-peptide aggregation. Neurosci. Lett..

[198] Caletti G., Almeida F.B., Agnes G., Nin M.S., Barros H.M.T., Gomez R. (2015). Antidepressant dose of taurine increases mRNA expression of GABAA receptor α2 subunit and BDNF in the hippocampus of diabetic rats. Behav. Brain Res..

[199] Caletti G., Herrmann A.P., Pulcinelli R.R., Steffens L., Morás A.M., Vianna P., Chies J., Moura D.J., Barros H.M.T., Gomez R. (2017). Taurine counteracts the neurotoxic effects of streptozotocin-induced diabetes in rats. Amino Acids.

[200] Agca C.A., Tuzcu M., Hayirli A., Sahin K. (2014). Taurine ameliorates neuropathy via regulating NF-κB and Nrf2/HO-1 signaling cascades in diabetic rats. Food Chem. Toxicol..

[201] Rahmeier F.L., Zavalhia L.S., Tortorelli L.S., Huf F., Géa L.P., Meurer R.T., Machado A.C., Gomez R., Fernandes M.D.C. (2016). The effect of taurine and enriched environment on behaviour, memory and hippocampus of diabetic rats. Neurosci. Lett..

[202] Li K., Shi X., Luo M., Llah I.-U., Wu P., Zhang M., Zhang C., Li Q., Wang Y., Piao F. (2019). Taurine protects against myelin damage of sciatic nerve in diabetic peripheral neuropathy rats by controlling apoptosis of schwann cells via NGF/Akt/GSK3β pathway. Exp. Cell Res..

[203] Garcia-Serrano A.M., Vieira J.P.P., Fleischhart V., Duarte J.M.N. (2022). Taurine or *N*-acetylcysteine treatments prevent memory impairment and metabolite profile alterations in the hippocampus of high-fat diet-fed female mice. bioRxiv.

[204] El Idrissi A., El Hilali F., Rotondo S., Sidime F. (2017). Effects of Taurine Supplementation on Neuronal Excitability and Glucose Homeostasis. Adv. Exp. Med. Biol..

[205] Ribeiro R.A., Santos-Silva J.C., Vettorazzi J.F., Cotrim B.B., Mobiolli D.D.M., Boschero A.C., Carneiro E.M. (2012). Taurine supplementation prevents morpho-physiological alterations in high-fat diet mice pancreatic beta-cells. Amino Acids.

[206] Drabkova P., Sanderova J., Kovarik J., Kandar R. (2015). An Assay of Selected Serum Amino Acids in Patients with Type 2 Diabetes Mellitus. Adv. Clin. Exp. Med..

[207] Lee S., Olsen T., Vinknes K.J., Refsum H., Gulseth H.L., Birkeland K.I., Drevon C.A. (2018). Plasma Sulphur-Containing Amino Acids, Physical Exercise and Insulin Sensitivity in Overweight Dysglycemic and Normal Weight Normoglycemic Men. Nutrients.

[208] Anuradha C.V., Balakrishnan S.D. (1999). Taurine attenuates hypertension and improves insulin sensitivity in the fructose-fed rat, an animal model of insulin resistance. Can. J. Physiol. Pharmacol..

[209] Nakaya Y., Minami A., Harada N., Sakamoto S., Niwa Y., Ohnaka M. (2000). Taurine improves insulin sensitivity in the Otsuka Long-Evans Tokushima Fatty rat, a model of spontaneous type 2 diabetes. Am. J. Clin. Nutr..

[210] Nandhini A.T.A., Anuradha C.V. (2002). Taurine modulates kallikrein activity and glucose metabolism in insulin resistant rats. Amino Acids.

[211] Camargo R.L., Branco R.C.S., De Rezende L.F., Vettorazzi J.F., Borck P.C., Boschero A.C., Carneiro E.M. (2015). The Effect of Taurine Supplementation on Glucose Homeostasis: The Role of Insulin-Degrading Enzyme. Adv. Exp. Med. Biol..

[212] Borck P.C., Vettorazzi J.F., Branco R.C.S., Batista T.M., Santos-Silva J.C., Nakanishi V.Y., Boschero A.C., Ribeiro R.A., Carneiro E.M. (2018). Taurine supplementation induces long-term beneficial effects on glucose homeostasis in ob/ob mice. Amino Acids.

[213] Vettorazzi J.F., Ribeiro R.A., Borck P.C., Branco R.C.S., Soriano S., Merino B., Boschero A.C., Nadal A., Quesada I., Carneiro E.M. (2016). The bile acid TUDCA increases glucose-induced insulin secretion via the cAMP/PKA pathway in pancreatic beta cells. Metabolism.

[214] Ito T., Schaffer S.W., Azuma J. (2011). The potential usefulness of taurine on diabetes mellitus and its complications. Amino Acids.

[215] Brøns C., Spohr C., Storgaard H., Dyerberg J., Vaag A.A. (2004). Effect of taurine treatment on insulin secretion and action, and on serum lipid levels in overweight men with a genetic predisposition for type II diabetes mellitus. Eur. J. Clin. Nutr..

[216] Schlesinger S., Neuenschwander M., Barbaresko J., Lang A., Maalmi H., Rathmann W., Roden M., Herder C. (2021). Prediabetes and risk of mortality, diabetes-related complications and comorbidities: Umbrella review of meta-analyses of prospective studies. Diabetology.

[217] Livingston G., Huntley J., Sommerlad A., Ames D., Ballard C., Banerjee S., Brayne C., Burns A., Cohen-Mansfield J., Cooper C. (2020). Dementia prevention, intervention, and care: 2020 report of the Lancet Commission. Lancet.

[218] Guerreiro R.J., Gustafson D.R., Hardy J. (2012). The genetic architecture of Alzheimer’s disease: Beyond APP, PSENs and APOE. Neurobiol. Aging.

[219] Albanese E., Launer L.J., Egger M., Prince M., Giannakopoulos P., Wolters F.J., Egan K. (2017). Body mass index in midlife and dementia: Systematic review and meta-regression analysis of 589,649 men and women followed in longitudinal studies. Alzheimer’s Dementia Diagn. Assess. Dis. Monit..

[220] Qizilbash N., Gregson J., Johnson M., Pearce N., Douglas I., Wing K., Evans S., Pocock S.J. (2015). BMI and risk of dementia in two million people over two decades: A retrospective cohort study. Lancet Diabetes Endocrinol..

[221] Sun S., He D., Luo C., Lin X., Wu J., Yin X., Jia C., Pan Q., Dong X., Zheng F. (2022). Metabolic Syndrome and Its Components Are Associated with Altered Amino Acid Profile in Chinese Han Population. Front. Endocrinol..

[222] Li Y.-C., Li Y.-Z., Li R., Lan L., Li C.-L., Huang M., Shi D., Feng R.-N., Sun C.-H. (2018). Dietary Sulfur-Containing Amino Acids Are Associated with Higher Prevalence of Overweight/Obesity in Northern Chinese Adults, an Internet-Based Cross-Sectional Study. Ann. Nutr. Metab..

